# Heterosis and Combining Ability Estimates in Isoflavone Content Using Different Parental Soybean Accessions: Wild Soybean, a Valuable Germplasm for Soybean Breeding

**DOI:** 10.1371/journal.pone.0114827

**Published:** 2015-01-21

**Authors:** Yingdong Bi, Wei Li, Jialei Xiao, Hong Lin, Ming Liu, Miao Liu, Xiaoyan Luan, Bixian Zhang, Xuejun Xie, Donglin Guo, Yongcai Lai

**Affiliations:** 1 Heilongjiang Academy of Agricultural Sciences Postdoctoral Programme, Northeast Forestry University Postdoctoral Programme, Heilongjiang Academy of Agricultural Sciences, 368 Xuefu Road, Harbin, 150086, China; 2 Institute of Crops Tillage and Cultivation, Heilongjiang Academy of Agricultural Sciences, 368 Xuefu Road, Harbin, 150086, China; 3 Institute of Crops Breeding, Heilongjiang Academy of Agricultural Sciences, 368 Xuefu Road, Harbin, 150086, China; 4 Institute of Soybean, Heilongjiang Academy of Agricultural Sciences, 368 Xuefu Road, Harbin, 150086, China; 5 Institute of Food Processing, Heilongjiang Academy of Agricultural Sciences, 368 Xuefu Road, Harbin, 150086, China; 6 Life Science and Technology College, Harbin Normal University, No.1 South Normal University Road, Limin Economic and Technological Development Zone, Harbin, 150025, China; Nanjing Agricultural University, CHINA

## Abstract

Isoflavone, a group of secondary metabolites in soybean, is beneficial to human health. Improving isoflavone content in soybean seeds has become one of the most important breeding objectives. However, the narrow genetic base of soybean cultivars hampered crop improvement. Wild soybean is an extraordinarily important gene pool for soybean breeding. In order to select an optimal germplasm for breeding programs to increase isoflavone concentration, 36 F1 soybean progenies from different parental accessions (cultivars, wild, Semi-wild and Interspecific) with various total isoflavone (TIF) concentration (High, Middle, Low) were analyzed for their isoflavone content. Results showed that male parents, except for Cultivars, showed positive GCA effects. In particular, wild soybean had higher positive GCA effects for TIF concentration. Both MP and BP heterosis value declined in the hybrid in which male parents were wild soybean, semi-wild soybean, interspecific offspring and cultivar in turn. In general, combining ability and heterosis in hybrids which had relative higher TIF concentration level parents showed better performance than those which had lower TIF concentration level parents. These results indicated characteristics of isoflavone content were mainly governed by additive type of gene action, and wild relatives could be utilized for breeding of soybean cultivars with this trait. A promising combination was found as the best potential hybrid for isoflavone content improvement.

## Introduction

Soybean, *Glycine max (L.) Merr.*, is the world’s most important oilseed crop. Isoflavone belongs to a group of secondary metabolites which are an important class of compounds that mediate multiple plant-microbial interactions in soybean [1]. Isoflavone consumption is also associated with human health benefits such as decreased risk of heart disease, reduced risk of prostate cancer or cardiovascular disease [2–5]. Studies have shown large variations in isoflavone concentrations and compositions among soybean genotypes [6–8]. In soybean, isoflavone accumulation and composition are affected by environmental conditions [9–11] and genotype [12–14]. According to the previous reports, genotype significantly influences the content and composition of isoflavones in soybean seeds and the potential for isoflavone production is largely under genetic control [15,16]. Because of benefits to food and human health, breeding soybean seeds with a desirable isoflavone content has become one of the most important breeding objective [17,18]. However, the narrow genetic base of soybean cultivars hinders crop improvement [19]. Using heterosis may be one strategy to increase TIF concentration in soybean cultivars and to meet the demand generated by the functional food market. Liang et al.(2005) reported that soybean cultivars, with high isoflavone levels, should be used as one of the parents in the breeding program, and it was the best choice to use combinations that were crossed between two high isoflavone varieties in North Carolina II mating design, in eight soybean cultivars [20]. Sun et al.(2002) found that there was heterosis in F1, F2 generations of most combinations among fifteen combinations with six soybean cultivars of different isoflavone contents. They predicted the selection might be carried out preliminarily in F2 hybrids [21]. However, soybean narrow genetic base make high isoflavone breeding very difficult. The exotic germplasm offers a vast genetic resource that can broaden soybean’s genetic base. The wild soybean (*Glycine soja, ET Zucc.*) is commonly accepted as the ancestral species of the cultivated soybean (*G. max (Linn.) Merr.*) [22]. Serving as valuable genetic resources, the wild soybean gene pool is extraordinarily important for soybean breeding, particularly when the genetic background of the cultivated soybean becomes narrow under the extensive modern agricultural practices [23,24]. In our previous study, we determined total isoflavone (TIF) concentration from 60 Chinese soybean accessions including 28 wild soybean (*Glycine soja*) and 32 cultivated soybean (*Glycine max*). The isoflavone levels in these accessions vary greatly, ranging from 461.2–6808.2μg/g dry seed weight [25]. Previously, we attempted to manipulate the exotic germplasm to improve the soybean total isoflavone (TIF) concentration. Lai proposed that crossing between high isoflavone wild soybean and high isoflavone cultivated soybean would enhance a rapid progress in the breeding of high isoflavone soybeans [26].

Overall, some researches on genetic control isoflavone concentration in soybean have been performed which provided useful information about usefulness of the parents in breeding programs [14,18,27]. However, the law of inheritance of isoflavone levels is not well known, especially the hybrid combination among different species such as cultivar or wild soybean. In hybrid oriented breeding programs, the knowledge of combining ability of the parents and the traits inheritance of is important [28,29]. The breeder can make use of this information to find the best strategy to select desirable parents or determine which breeding procedure will efficiently improve the performance of the traits of interest [30,31]. The objective of this study was to determine the general and specific combining ability of 15 different soybean species (cultivars, wild, Semi-wild and Interspecific) and their 36 hybrid combinations, as well as to examine the heterosis on isoflavone concentration. We also aimed to improve parental selection and germplasm for breeding programs to increase isoflavone concentration.

## Materials and Methods

### Germplasm

To determine the general and specific combining ability of different soybean species, A total of 15 accessions including cultivars, interspecific offsprings and wild relatives were used here. The interspecific offsprings were selected from artificial hybrid offsprings between wild and cultivated soybeans. The wild relatives were classified as typical wild or semi-wild soybeans according to Wang et al. (2012). The 100-kernel weight of typical wild soybean is below 3.0 g, whereas semi-wild soybean is over 3.0 g. The 3 cultivars lines were designated as females and crossed to 12 different accessions as males in accordance with incomplete diallel cross to generate 36 hybrids.12 male parents were divided into four groups which are cultivars, wild, semi-wild and interspecific offspring.

The contents of 4 main derivatives of isoflavone (Daidzin, Daidzein, Genistin, Genistein) were determined by HPLC. As a result, the total content of isoflavone (Daidzin, Daidzein, Genistin, Genistein) showed significant difference especially between the H group and the M group levels. Each group of male parents comprised 3 lines each based on the isoflavone (TIF) concentration of their dry seed which is High (4283∼5618μg/g), Middle(2281∼3858μg/g) and Low (848∼1717μg/g) as well as female parents. The males were constituted from introduced and Heilongjiang province (collected Germplasms), while female parents were obtained from the Crops Breeding Institute of Heilongjiang Academy of Agricultural Sciences ( HAAS ) in China (Table 1).

**Table 1:** Origin and pedigree of parental soybean lines used to generate 36 F1 hybrids in this study.

**Entry number**	**Name**	**Origin**	**Pedigree**	**Species group**	**Isoflavone Content Classification**	**Isoflavone Content (μg/g)**
**Female**						
**1**	Longpin 05-277	CBI HAAS	unknown	Cultivar	H	3955±142
**2**	Hefeng 47	CBI HAAS	Hefeng35×Gong84112-1-3	Cultivar	M	2341±119
**3**	Heinong 35	CBI HAAS	Heinong16×SSCY	Cultivar	L	1341±58
**Male**						
**1**	W01-177	GBWS HAAS	Glycine soja	Wild soybean	H	3682±111
**2**	W01-355	GBWS HAAS	Glycine soja	Wild soybean	M	2371±125
**3**	W01-694	GBWS HAAS	Glycine soja	Wild soybean	L	1770±24
**4**	W01-491	CBI HAAS	Landrace	Semi-wild soybean	H	4649±289
**5**	W01-175	CBI HAAS	Landrace	Semi-wild soybean	M	3126±195
**6**	W01-555	CBI HAAS	Landrace	Semi-wild soybean	L	938±88
**7**	Longpin01-122	CBI HAAS	Strain	Interspecific offspring	H	3583±102
**8**	Longpin05-67	CBI HAAS	Strain	Interspecific offspring	M	2116±67
**9**	ZYD 5531	CBI HAAS	Strain	Interspecific offspring	L	1668±13
**10**	Heinong37	SRI HAAS	H77-7594×H78-8391	Cultivar	H	3397±173
**11**	Hefeng39	HI HAAS	87-1004×Hejiao87-19	Cultivar	M	2673±54
**12**	Long05-372	CBI HAAS	unknown	Cultivar	L	1961±76

### Field experiments

Feild experiments were performed at Minzhu Experimental plot of Heilongjiang Academy of Agricultural Sciences (HAAS). The park management committee of HAAS authorized the permission for these experiments. The experiment was laid out as a row-column a-design with three replications at the Minzhu Experimental plot (45.8457 N, 126.8501 E), Harbin, China from 2009 to 2010. Female and male parents were crossed according to the experimental design to generate 36 F1 hybrids in 2009. Seeds of all parents and hybrids were planted by hand in one-row plots of 5.0 m length at 0.75m inter-row and 0.15 m intra-row spacing in 2010. The seeds were harvested oven dried (14% moisture content) and kept in air conditioned room (23°C ±2).

### Isoflavones determination

Approximately 20 g of seed was dried and ground in a high speed (20,000 rpm) mill for 25 s (Knifetec Sample Mill; FOSS). A total of 2g powder was mixed with 10 ml of 80% ethanol, and the mixture was sonicated for 1 h at 50°C to extract constituents before filtering through a 0.45μm syringe filter. In total, 153 samples (three replications) were analyzed for isoflavone concentration by high performance liquid chromatography (HPLC) using the modified methods of Wang and Murphy [32] and Kim, Jung, Ahn, and Chung [33].A Warters 600 HPLC system, together with Waters 2996 Photo Diode Array Detector (Milford,Massachusetts, USA) equipped with a YMC Symmetry C18 5μm 4.6×250mm Column and a Waters 2707 Auto injector were employed. The wavelength of the UV detector was 254 nm. A linear HPLC gradient was engaged using solvent A (methanol) and solvent B (distilled water). Following the injection of 20 μL sample, solvent B was increased from 20% to 90% in 30 min, kept at 90% for 15 min, and returned to 20% in 1 min at the flow rate of 0.5 ml/min. After a total lapse of 50 min, equivalent treatment of the next sample was commenced.

### Data analyses

The data was subjected to analysis of variance(ANOVA) by the General Linear Model (GLM) and the analysis of variance procedures using SPSS software. The form of the combining ability analysis of variance employed was Model 2, Method 1 of Griffing (1956) as presented by Singh (1973):

Xijl=μ+gi+gj+sij+sji+(gl)i+(gl)j+(sl)ij+1/bcεεeijmr

where: Xijl = Mean of ixj genotype; gi = General combining ability (GCA) effect of the ith parent; gj = General combining ability (GCA) effect of jth parent; sij = Specific combining ability (SCA) effect of i × jth cross;(gl)i = Interaction of GCA effect of ith parent with;(gl)j = Interaction of GCA effect of jth parent sji = Reciprocal effect of j × ith cross; (sl)ij = SCA effect of i × jth cross;1/bcεεeijkmr = Mean error effect.

GCA and SCA effects were estimated, respectively (Singh, 1973), as follows:

ĝi=(nxi..−2X…)/n(n−2)

Ŝij=(Xij.)−[(Xi..+Xj..)/(n−2)]+2X/(n−1)(n−2)

Heterosis: Using means computed from the combined analysis, percentage heterosis based on mid-parent (MP) and better parent (BP) values were been calculated according to the formula, using the following formula described by Davis(1978) as follows:

Mid-parent heterosis(MPH)=(F1−((P1+P2)/2)×100/(P1+P2)/2

Better-parent heterosis(BPH)=(F1−BP)×100/BP

Where: F1 = the mean of F1 hybrid

P1, P2 and BP = means of the first, the second and better parent respectively.

## Results

### Mean performance and Analysis of variance

**Figure 1:**
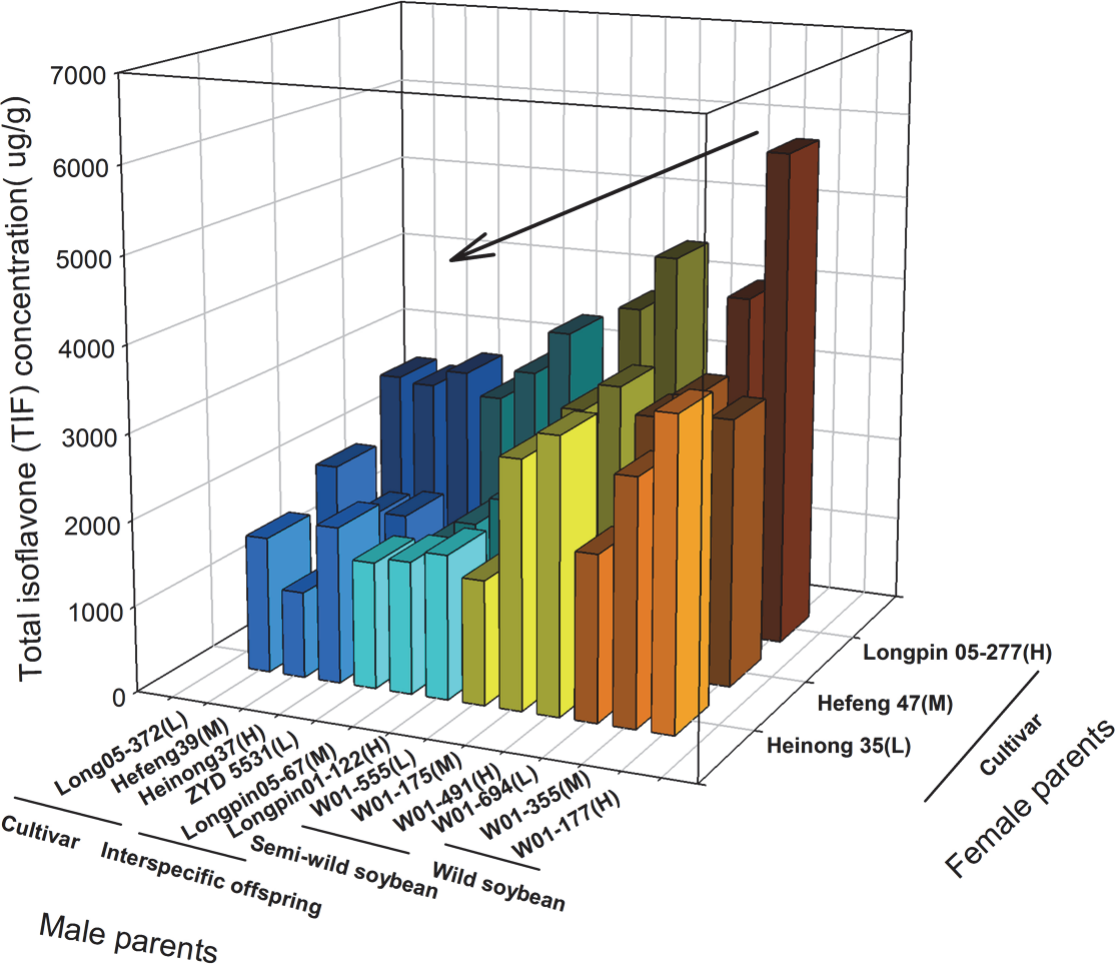
Average total isoflavone (TIF) concentration (μg/g) of hybrids gained by fifteen parents. Male parents were divided into four groups which are cultivars, wild, Semi-wild and Interspecific offspring based on plant taxonomy. Female parents comprised 3 lines each based on TIF concentration which is H (high), M (middle) and L (low).

Mean total isoflavone (TIF) concentration of hybrids vary greatly, ranging from as low as 1003 μg/g of hybrid Heinong 35×Hefeng 39 to 5822 μg/g of the hybrid Longpin 05-277×W01-177. The nine hybrids which have wild soybean as male parents had highest average TIF (3397.2μg/g) followed by semi-wild soybean (2892.10 μg/g), Interspecific offspring (2023.7μg/g) then the Cultivar (1947.0 μg/g) which had the lowest average TIF. The mean total isoflavone concentration were relatively high in twelve hybrids which have a female parent classified High TIF, compared with those middle and low TIF classified hybrids. Furthermore, among the combinations that were crossed between male parents in a species group and the same female parent, the mean TIF concentration showed a decreasing trend in hybrids whose male parents were classified from high to low TIF (Fig. 1).The analysis of variance (ANOVA) revealed highly significant differences (P< 0.01) among genotypes for TIF concentration, The mean squares of parents and crosses were significantly different at a 1% level of probability. The difference in TIF concentration between different combinations indicated that they are suitable for genetic studies. No significant differences were observed among the different ICC (Isoflavone Content Classification) values of male parents. Highly significant differences were, however, observed among different species of male parents (Table 2). These results indicates the presence of sufficient genetic variability among genotypes which can be exploited in a soybean breeding program for improving total isoflavone (TIF) concentration.

**Table 2:** Mean squares from the combined analysis of variances for Isoflavone Content for Parents.

**Female**		**N**	**Subset**	
			**1**	**2**
Isoflavone Content Classification				
Duncan(a,b)				
	low	12	2084.81	
	middle	12	2236.53	
	high	12		3373.70
Male	Sig.		.668	1.000
Species				
Duncan(a,b)				
	cultivar	9	1947.03	
	interspecific	9	2023.72	
	semi-wild	9		2892.10
	wild	9		3397.19
			.830	.165

Means for groups in homogeneous subsets are displayed. Based on Type III Sum of Squares, The error term is Mean Square(Error) = 732156.927.

^a^ Uses Harmonic Mean Sample Size = 12.000.

^b^ Alpha = .05.

### Combining ability effects

The magnitudes of GCA and SCA effects are indicative of the relative importance of additive and non-additive gene actions in the inheritance of a trait, respectively. The large GCA: SCA variance ratio suggests the importance of additive gene effects, whereas a low ratio signifies presence of dominant and/or epistatic gene effects [34]. The mean squares of both GCA and SCA were highly significant due to both male and female parents for the TIF (Table 3). Moreover, a higher σ^2^_g_/σ^2^_s_ ratio (75:25) was observed in this syudy, which indicates that the predominance of additive gene action is more important for TIF. Heritability is a key parameter in quantitative genetics because it determines the response to selection [31]. A higher narrow sense heritability of 70.42% was exhibited by TIF which suggested that TIF has a high transmitting ability to the next generation (Table 3). This result implies that genes with additive effects were important for these traits and breeding progress could be achieved through selection of good parents.

**Table 3:** Mean squares from the combined analysis of variances for the Isoflavone content measured.

**Source of variation**	**Type III Sum of Squares**	**df**	**Mean Square**	**F**	**Sig.**
Replications	95347.62	2	47673.81	0.6898	0.5051
Genotypes	116936974	35	3341056	48.3402**	0.0001
Female	18565000	2	9282500	9.3866**	0.0011
Male	76615947	11	6965086	7.0432**	0.0001
Female×Male	21756028	22	988910.4	14.3081**	0.0001
Male ICC (Isoflavone Content Classification)	6188333.317	2	3094166.659	4.226*	.025
Male S (Species)	13269642.095	3	4423214.032	7.898**	.001
Female ICC×Male ICC	1112698.351	4	278174.588	.380	.821
Female ICC×Male S	359283.949	6	59880.658	.107	.995
Error	4838080	70	69115.43		
Total	121870402	107			
					
Variance component estimates					
δ^2^_f_	664019.5204				
δ^2^_m_	230377.4892				
δ^2^_fm_	306598.3104				
δ^2^_e_	69115.4336				
Proportional contribution to total variances					
δ^2^_f_+δ^2^_m_/δ^2^_G_	74.47%				
δ^2^_fm_/δ^2^_G_	25.53%				
Broad Sense heritability (h^2^b)	94.56%				
Narrow Sense heritability (h^2^n)	70.42%				

df = degrees of freedom.

* Significant at P<0.05.

** Significant at P<0.01.

The GCA effects for male parents were highly variable in the different species. Positive GCA effects were shown in twelve male parents, three wild soybeans, three semi-wild soybeans and one interspecific offspring (ZYD 5531). In particular, wild soybean had higher positive GCA effects for TIF concentration. however, cultivars, showed negative GCA effects. Positive GCA effects were shown in two female parents, Longpin 05-277 and Hefeng 47, but not Heinong 35. W01-177 was found to be the best general combiner by having maximum GCA effects (65.26) for TIF followed by W01-355 (38.24) and W01-175 (23.33) (Table 4). Twenty specific crosses showed positive SCA effects ranging from 0.25 to 127.01, while maximum SCA effects were gained by the hybrids Longpin 05-277 (H TIF) × W01-177 (wild soybean H TIF) followed by Longpin 05-277 (H TIF) × W01-355 (wild soybean M TIF) and Hefeng 47(M TIF) × high and W01-177 (wild soybean H TIF).

**Table 4:** Estimates of general combining ability (GCA) effects for the 15 parents of soybean species and specific combining ability (SCA) effects for Isoflavone Content for the 36 F1 hybrids across.

**Parents**			**Female (Cultivar)**			
						
**Male**			**H**	**M**	**L**	
			Longpin 05-277	Hefeng 47	Heinong 35	GCA values
**Wild soybean**	H	W01-177	42.4635	−6.864	−35.5995	65.2636
	M	W01-355	17.4661	10.3447	−27.8107	38.2429
	L	W01-694	−0.991	−3.1401	4.1312	16.391
**Semi-wild soybean**	H	W01-491	−14.7808	−4.3561	19.1369	6.2133
	M	W01-175	−21.2601	5.593	15.667	23.3311
	L	W01-555	9.0768	14.8707	−23.9475	2.0995
**Interspecific offspring**	H	Longpin01-122	−6.3931	−1.2654	7.6585	−40.7505
	M	Longpin05-67	−24.9118	−8.9175	33.8293	−35.9058
	L	ZYD 5531	12.0417	1.2942	−13.3358	8.7361
**Cultivar**	H	Heinong37	7.6191	13.608	−21.227	−2.0869
	M	Hefeng39	−14.8103	−1.9045	16.7149	−38.9488
	L	Long05-372	−5.5198	−19.263	24.7828	−42.5856
		GCA values	19.2844	0.9876	−20.272	

### Heterosis and Better parent heterosis

**Figure 2:**
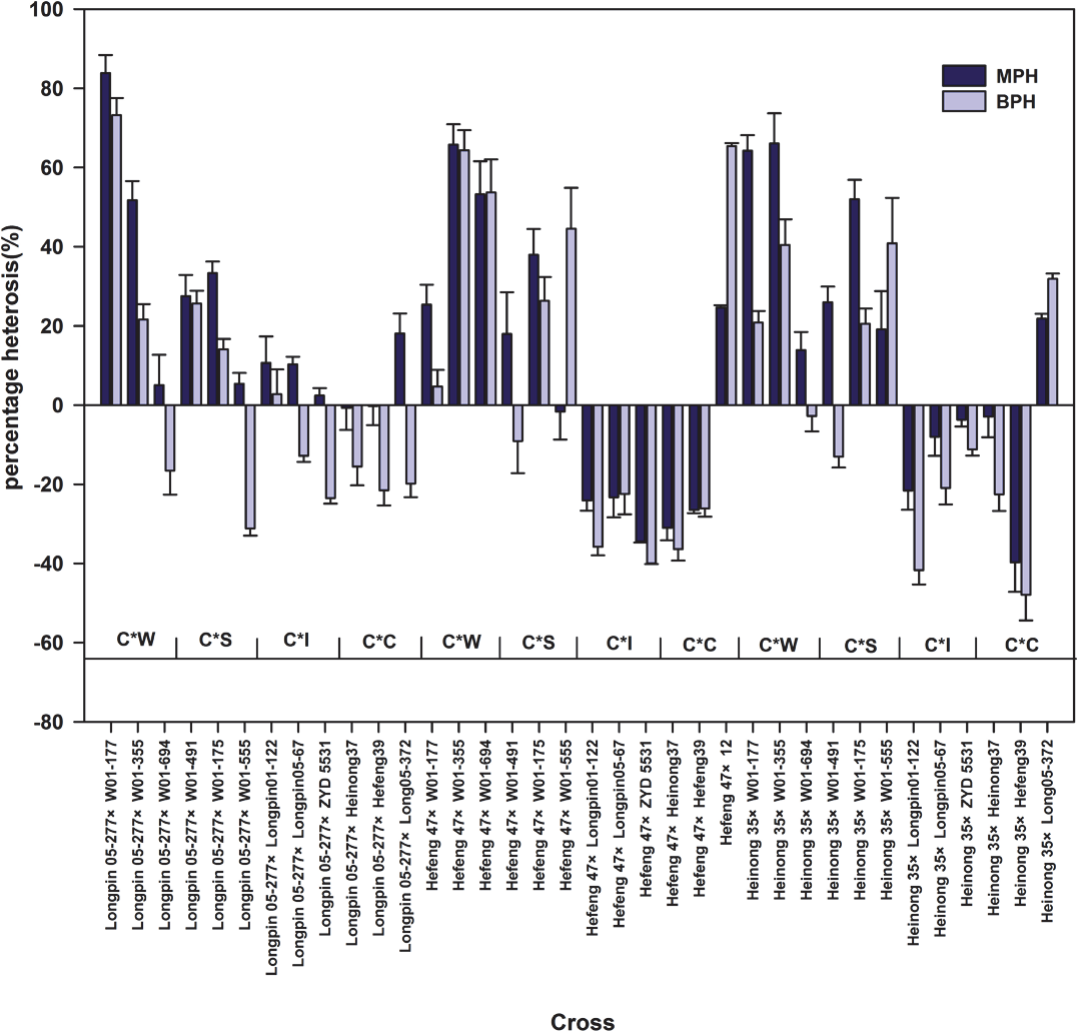
Estimates of mid parents (MP) and better parent (BP) heterosis (%) for total isoflavone (TIF) concentration in 36 hybrids. Combinations were classified based on plant taxonomy of parents. C cultivars, W wild, S semi-wild, I interspecific offspring.

The heterosis percentages values relative to midparents (MP) and better parent (BP) are presented in Fig. 2. All the combinations showed a different degree of heterosis for TIF. The midparents’ heterosis ranged from −39.71 to 83.88% for the cross Heinong 35 × Hefeng 39 and Longpin 05-277 × W01-177. Better parents’ heterosis ranged from −47.92 to 73.25% for the cross Heinong 35 × Hefeng 39 and Longpin 05-277 × W01-177. Twenty-three hybrids displayed positive midparents heterosis and 16 hybrids had positive better parents heterosis value for TIF. Most of the crosses which male parents are wild soybean or semi-wild soybean showed a high positive heterosis and a positive better parent heterosis for TIF. However, a low heterosis was observed for the crosses in which male parents are interspecific offspring or cultivar.

The Longpin 05-277 × W01-177 cross showed the maximum midparents heterosis value of 83.88% as well as the maximum better parents heterosis value of 73.25% for TIF. The relatively narrow range of 2.4 to 10.8% in midparent heterosis was observed among the Longpin 05-277 × Longpin01-122, Longpin 05-277 × Longpin05-67and Longpin 05-277 × ZYD 5531 combinations with different TIF of female parents (High, Middle and Low), whereas the widest range of −16.51 to 73.25% in better parent heterosis was observed among combinations the Longpin 05-277 × W01-177, Longpin 05-277 × W01-355 and Longpin 05-277 × W01-694 combinations.

## Discussion

### Genetic effects of isoflavone concentration

Significant GCA and SCA mean squares values were observed in the parents and hybrids, respectively, and showed that both additive and non-additive gene effects played a role in TIF accumulation. However, the large GCA variance was more predominant than the SCA indicating that additive gene action was more important than non-additive gene action in hybrids. Significant mean squares for GCA and SCA in the isoflavone contents and its components have previously been reported, Liang et al. suggested that a soybean variety with high isoflavone levels should be used as one of the parents in the breeding program. However, no significant differences were observed among the different ICC (Isoflavone Content Classification) of male parents in our research. To identify the genetic effects that underlie the isoflavone content in soybean seeds, four groups (cultivars, wild, Semi-wild and Interspecific offspring) were used as male parents to estimate their combining ability. Highly significant differences were, however, observed among different species of male parents, Comparisons between GCA effects (gi) associated with individual parents in TIF content are illustrated in Table 4. These results revealed that the wild soybean had higher positive GCA effects than the other three species in total isoflavone (TIF) concentration. Crop wild relatives (CWRs) will gain an importance in plant breeding to produce new varieties [35–37]. As a result of the crop improvements made using wild germplasm, crop breeders and researchers have been encouraged to further exploit wild genomes in search of valuable traits [38–40]. Some attempts to broaden the genetic base of soybeans by utilizing G. soja were reported and some genetically stable lines which showed significantly improved disease resistance were obtained in crosses between G. soja and G. max [41–44]. In our research, three wild soybean male parents showed higher GCA effects, moreover, maximum SCA effects were gained by the Longpin 05-277 (H TIF) × W01-177 (wild soybean H TIF) hybrids indicating that the valuable breeding material discovered through wild soybean exploitation.

Significant difference between MP and BP heterosis were recorded among 36 crossing combinations. The analysis of heterosis revealed a significant trend in hybrids based on species or the parents’ TIF concentration. Relatively higher heterosis or better parent heterosis for TIF were observed in hybrids in which male parents are wild soybean followed by semi-wild soybean, interspecific offspring and cultivar,While a downward trend was detected in combinations from high to low TIF female parent. Moreover, the highest heterotic performance for TIF was recorded in Longpin 05-277 (H TIF) × W01-177 (wild soybean H TIF) hybrid. These observations suggested that the wild relatives and high TIF phenotypes could be exploited as genetic resources for soybean isoflavone content improvement. Li et al. illustrated that the G. Soja allele at Satt511 on LG A1 was associated with increased seed yield, and this result demonstrated the potential of identifying positive alleles in the exotic germplasm of soybean [45]. A related interspecific phenomenon has been reported in alfalfa [46], Common bean [47,48],cotton [49], rice [50] and Wheat [51], Many investigators proposed that favorable heterosis varied according to the cross combinations for different traits in soybean [43–45,52].

### Selection strategies for total isoflavone (TIF) concentration varieties

Wild relatives of crop species are often sources of genes for disease resistance, increased yield and improved product quality or fitness [53]. Wild forms and closely related species of wild soybean, therefore, have great potential as an additional source of useful germplasm for soybean improvement [54,55]. For decades, but more intensely in recent years, efforts have been made for soybean improvement by using wild relatives for yield [56], fitness [43], protein [57] and disease resistance [58]. Isoflavone improvement has been a promising target in soybean breeding program for favorable health promoting characteristics [59,60]. While heterosis and combining ability analysis for Isoflavone content in wild soybeans is poorly documented, some genetic effects that underlie the isoflavone content in soybean seeds have been identified [20]. In our study, we found that combining ability and heterosis of wild species were significantly better than cultivar or other species. Jelena Cvejić et al. revealed that isoflavone content is the trait that can be derived from parent genotype to its F1 progenies [61]. Interestingly, the highest SCA and heterotic performance for TIF was gained in Longpin 05-277 (H TIF) × W01-177 (wild soybean H TIF) hybrid. Therefore,these F1 progenies could be exploited as genetic resources for breeding of specific isoflavone characteristic. However, much more research is required to meet breeding objective due to complex selection processes. Jorge A.Acosta-Gallegos proposed that prebreeding efforts will be enhanced by information on gene pool origins, domestication syndrome traits, mapping data of the wild forms and marker-assisted selection [48].

## Conclusions

In this study, 36 F1 soybean progenies were analyzed for their isoflavone content using different species’parental lines (cultivars, wild, semi-wild and interspecific) which were divided into 3 levels each based on the isoflavone (TIF) concentration of their dry seed. Our results showed that most of male parents, except for cultivars, showed positive GCA effects. In particular, wild soybean had higher positive GCA effects for TIF concentration.Similarly, both MP and BP heterosis decreased in hybrids which male parents were wild soybean, semi-wild soybean,Interspecific offspring and cultivar in turn. In general, combining ability and heterosis in hybrids which had higher TIF level parents showed better performance than those had middle or low level. These results indicated characteristic of isoflavone content were mainly governed by additive type of gene action and wild relatives could be utilized for breeding of soybean cultivars with these characteristics. Moreover, a promising combination of Longpin 05-277 (H TIF)×W01-177 (wild soybean H TIF) demonstrated the best potential hybrid for isoflavone content followed by Longpin 05-277 (H TIF) ×W01-355 (wild soybean M TIF). Our results also suggested such promising hybrids could be used in breeding of soybean for improved isoflavone content enhanced by marker-assisted selection.
